# Disintegration of wheat aleurone structure has an impact on the bioavailability of phenolic compounds and other phytochemicals as evidenced by altered urinary metabolite profile of diet-induced obese mice

**DOI:** 10.1186/1743-7075-11-1

**Published:** 2014-01-02

**Authors:** Jenna Pekkinen, Natalia N Rosa, Otto-Ilari Savolainen, Pekka Keski-Rahkonen, Hannu Mykkänen, Kaisa Poutanen, Valérie Micard, Kati Hanhineva

**Affiliations:** 1 Institute of Public Health and Clinical Nutrition, Clinical Nutrition, University of Eastern Finland, Kuopio Campus, P.O. Box 1627, Kuopio FI-70211, Finland; 2 JRU Agropolymers Engineering and Emerging Technologies (IATE 1208) SupAgro-INRA-UM2-CIRAD, 2 Place Pierre Viala, Montpellier cedex 1 34060, France; 3 VTT Technical Research Centre of Finland, Espoo, Finland

**Keywords:** Arabinoxylan, Ferulic acid, Microbial metabolism, Non-targeted metabolomics, LC-MS, Metabolite profiling

## Abstract

**Background:**

Phenolic acids are covalently bound to the arabinoxylan fibre matrix of wheat aleurone layer. In order to be bioavailable they need to be released by endogenous or bacterial enzymes and absorbed within the intestinal lumen. The intestinal microbiota can metabolize phenolic acids and other food-born phytochemicals. However, the effect of structure of the cereal bran or aleurone layer on these processes is not comprehensively studied.

**Methods:**

The structure of aleurone layer was modified either by dry-grinding or by enzymatic treatments with xylanase alone or in combination with feruloyl esterase. Diet induced obese C57BL6/J mice were fed with high-fat diets containing either pure ferulic acid, or one of the four differentially treated aleurone preparations for 8 weeks. The diets were designed to be isocaloric and to have similar macronutrient composition. The urinary metabolite profiles were investigated using non-targeted LC-qTOF-MS-metabolomics approach.

**Results:**

The different dietary groups were clearly separated in the principal component analysis. Enzymatic processing of aleurone caused increased excretion of ferulic acid sulfate and glycine conjugates reflecting the increase in unbound form of readily soluble ferulic acid in the diet. The urinary metabolite profile of the diet groups containing native and cryo-ground aleurone was more intense with metabolites derived from microbial processing including hippuric acid, hydroxyl- and dihydroxyphenylpropionic acids. Furthermore, aleurone induced specific fingerprint on the urinary metabolite profile seen as excretion of benzoxazinoid metabolites, several small dicarboyxlic acids, and various small nitrogen containing compounds.

**Conclusions:**

The structural modifications on wheat aleurone fraction resulted in altered metabolism of aleurone derived phenolic acids and other phytochemicals excreted in urine of diet-induced obese mice.

## Background

Whole grain consumption has been consistently related to lower incidence of cardiovascular disease, obesity, diabetes, and cancer in epidemiological studies [1,2]. In addition to being excellent source of fibre, whole grains contain also rich variety of bioactive secondary compounds produced by the plant/grain [3]. These bioactive compounds are mainly located in the outer membranes of the grain, which are recovered in the bran during the milling process.

Aleurone is one of the outermost layers of the cereal grain [4]. It is composed of one single layer of cells located between the endosperm and seed coat. The main component of wheat aleurone cell wall is arabinoxylan (AX), which is a nonstarch polysaccharide constituted of a linear backbone of beta-(1,4)-linked xylose residues that can be substituted with one or two arabinose residues [5]. AX makes the aleurone rich in dietary fibre (DF), but also rich in bioactive phenolic compounds as the arabinose residues of AX are generally highly substituted with phenolic acid residues such as ferulic acid (FA), which is the major phenolic acid present in the aleurone layer. These phenolic compounds have been suggested to play a role in the beneficial health effects of diets rich in whole grain wheat [2]. Ferulic acid (FA) is indeed mostly responsible for the antioxidant effects of the wheat grain [6], and in particular bran [7]. In addition to a wide variety of phenolic compounds, the aleurone layer is a good source of minerals and vitamins, which are located intracellularly [3].

Prior to reaching the circulation and being able to exert its biological effects FA needs to be released from the surrounding grain or tissue matrix either by the intestinal mucosal enzymes or by the intestinal bacterial enzymes [8,9]. However, the complex structure of the aleurone cell layer can directly affect the bioaccessibility and further bioavailability of FA [10] and other compounds travelling through the digestive tract. This so called ‘matrix effect’ might have an influence on the physiological response to the compounds bound to AX.

The bioaccessibility of phenolic compounds present in wheat bran can be improved by reduction of particle size (increasing the specific surface area) via ultra-fine grinding and by solubilization of cell wall polysaccharides via enzymatic processing using xylanase and feruloyl esterases [7,11,12]. Both increase of particle size and the enzymatic release of FA moieties as free or conjugated to oligosaccharides, were shown to improve the *in vitro* antioxidant capacity as compared to the unprocessed aleurone [7,13] suggesting that these modifications might also have different bioeffects *in vivo*. However, there are no human or animal studies so far investigating the effect of the matrix structure on bioavailability of FA and other AX-bound phytochemicals of aleurone layer. In the first part of this study by Rosa et al. (Rosa N, Pekkinen J, Zavala K, Fouret G, Ayhan Kormaz A, Feillet-Coudray C, Atalay M, Hanhineva K, Mykkänen H, Poutanen K, Micard V: Enzymatic modification of wheat aleurone reduces body weight and metabolic risk factors of obesity in mice fed a high-fat diet. Submitted) we showed that enzymatically processed aleurone layer with increased amount of free FA produce beneficial health effects, i.e. reduced bodyweight, adipose tissue amount and fasting insulin and leptin levels in a high-fat diet induced obese mouse model. Here, in the second part of the study, the aim was to analyze the urinary metabolic fingerprint of the mice from the study by Rosa et al. using non-targeted LC-qTOF-MS metabolomics approach in order to elucidate the differences in bioavailable phytochemicals between the diets.

## Methods

### Wheat aleurone preparations and composition of diets

The wheat aleurone-rich fraction (A1) was provided by Bühler AG (Uzwil, Switzerland). Its chemical composition was 39.7 g dietary fibre, 22.2 g protein, 13.3 g ash, 9.8 g fat, and 5.8 g starch in 100 g aleurone dry matter. The processing of the aleurone was described in our previous work [13]. Shortly, following treatments were applied on the native aleurone (A1) to obtain three modified aleurone preparations: ultra-fine grinding with an impact mill in cryogenic conditions (A2), which produced an aleurone fraction with smaller particle size (1/3 of the native one); xylanase treatment (A3), which solubilized 43% of AX and released 17% of FA in a bioavailable form (conjugated plus free forms); successive xylanase and A-type feruloyl esterase (FAE) treatments (A4) which solubilized 82% of AX and released 87% of FA in bioavailable forms [14].

The details of the diets are described by Rosa et al. (Rosa N, Pekkinen J, Zavala K, Fouret G, Ayhan Kormaz A, Feillet-Coudray C, Atalay M, Hanhineva K, Mykkänen H, Poutanen K, Micard V: Enzymatic modification of wheat aleurone reduces body weight and metabolic risk factors of obesity in mice fed a high-fat diet. Submitted). Shortly, all the aleurone and commercial ferulic acid enriched diets were designed to match the commercial D12451 standard high-fat diet (45 E% of fat, Research Diets Inc., New Brunswick, NJ, USA) with calorie density, amount of macronutrients, and with total fibre content. Approximately 13% (w/w) of aleurone preparation (A1-A4) was added in the diets during the cold extrusion process by the manufacturer (Research Diets Inc., New Brunswick, NJ, USA). The energy and macronutrient distributions naturally present in the aleurone preparations were taken into account when planning the diets. Therefore, for instance, the total protein amount in the final aleurone diets consisted of the protein, naturally present in aleurone preparation and of the added commercial protein source, casein. The amount of commercial FA used in the FA control diet was designed to be similar with the FA amount in the final A1 diet (ca. 1 mg/g). The FA was added on the top of the diet, ie. nothing was removed.

### Animals and study design

The details of the study design and animal trial are described by Rosa et al. (Rosa N, Pekkinen J, Zavala K, Fouret G, Ayhan Kormaz A, Feillet-Coudray C, Atalay M, Hanhineva K, Mykkänen H, Poutanen K, Micard V: Enzymatic modification of wheat aleurone reduces body weight and metabolic risk factors of obesity in mice fed a high-fat diet. Submitted). Shortly, C57BL/6 J male mice were obtained from National Laboratory Animal Center (Kuopio, Finland) at the age of 9 weeks. The mice were acclimatized for one week and housed 3–4 per cage in regulated environment; temperature 22 ± 1°C, relative air humidity 55 ± 15% and 12/12 h light/dark cycle with lights on at seven AM. Due to increased occasions of fighting, the mice were separated to single cages from the week 13 until the end of the study (week 17). The mice were fed ad libitum a commercial high-fat diet (D12451, Research Diets Inc., USA) during weeks 1–9 to induce obesity and related metabolic disorders. After HFD prefeeding the mice were randomized into study groups (n = 9-14 per group) and were fed either with D12451 (high-fat diet control, HFD) or the D12451 diets enriched with aleurone fractions (A1-A4) or commercial FA for additional 8 weeks. The animal experiment was approved by The Institutional Animal Care and Use Committee of the Provincial Government of Finland.

### Urine sample preparation

Urine samples were collected on the week 17 directly into microcentrifuge tubes while the animals were handled. The number of urine samples per dietary group varied from two to four. One sample consists of a pool of urine from 3–4 mice housed in the same cage during the study. The urine samples were frozen on dry ice and stored at -80°C until thawed on ice bath and filtered using 0.2 μm Acrodisc^®^ Syringe Filters with a PTFE membrane (PALL Corporation, Ann Arbor, MI) prior subjecting to the LC-MS analyses.

### LC-MS conditions

Urine samples were analysed with Agilent UHPLC-qTOF-MS System using electrospray ionization (Jet Stream) in negative polarity (Agilent Technologies 1290 LC, 6540 MS). Chromatographic separations were performed using hydrophilic interaction chromatography (HILIC) with an Acquity UPLC BEH Amide column (100 mm × 2.1 mm, 1.7 μm; Waters Corporation, Milford, MA). Column temperature was 45°C, flow rate 0.6 ml/min, and the eluents A and B were 50% (v/v) and 90% (v/v) acetonitrile, respectively, both containing 20 mM of pH 3 ammonium formate. The gradient was as follows: 0–2.5 min: 100% B; 2.5–10 min: from 100% to 0% B; 10–10.01 min: from 0% to 100% B; 10.01–12.5 min: 100% B. Injection volume was 2 μl and sample tray temperature 4°C. Acetonitrile was from VWR International (Darmstadt, Germany). Ammonium formate and formic acid were from Sigma (Sigma-Aldrich, St. Louis, MO). All mobile phase constituents were of LC-MS or LC-MS Ultra grade. Water was purified using a Milli-Q Gradient system (Millipore, Milford, MA).

The ion source conditions were as follows: drying gas temperature 325°C and flow 10 L/min, sheath gas temperature 350°C and flow 11 L/min, nebulizer pressure 45 psi, capillary voltage 3500 V, nozzle voltage 1000 V, and fragmentor voltage 100 V. For data acquisition, 2 GHz extended dynamic range mode was used with an abundance threshold at 150, mass range of 20–1600, and scan time of 600 ms (1.67 Hz). For automatic MS/MS analyses, precursor isolation width was 1.3 Da, and from every precursor scan cycle, 4 ions with the highest intensity were selected for fragmentation. These ions were excluded after 2 product ion spectra and released after a 0.25-min hold. Precursor scan time was based on ion intensity, ending at 20000 counts or after 500 ms. Product ion scan time was 500 ms. Collision energies were 10, 20 and 40 V in subsequent runs. Continuous mass axis calibration was performed by monitoring two reference ions from an infusion solution throughout the runs (*m/z* 112.985587, *m/z* 966.000725). Data acquisition software was MassHunter Acquisition B.04.00.

### Non-targeted metabolomics data processing

Initial data processing was performed using MassHunter Qualitative AnalysisB.05.00 (Agilent Technologies, USA). Ions were extracted to compounds exhibiting isotopic peaks, dimers, and common adducts. The data were output as compound exchange format (.cef files) into the Mass Profiler Professional (MPP) software (version 2.2, Agilent Technologies) for compound alignment, statistical evaluation (ANOVA, student’s *t*-test), and chemometrics analysis by principal component analysis (PCA). In order to reduce noise and low abundance metabolites from the data matrix, only the metabolite features that appeared in at least 60% of samples in at least one of the dietary conditions having abundance at least 4.0E + 04 were retained in the data matrix, resulting in a dataset containing 3082 metabolite features. For PCA analyses, metabolite features were further filtered to satisfy a corrected q-value cut-off 0.05 (ANOVA, Benjamini-Hochberg false discovery rate correction for p-values) resulting in 662 entities. For the pairwise comparison of the diet groups, the data matrix (3082 features) was exported to Excel and filtered according to the abundance (n = 3 at least in one condition of FA or A1-A4), maximum average >4.0E + 05 in any of the groups FA or A1-A4 (in order focus on the most abundant peaks amenable for MS/MS fragmentation), q-value <0.05 (pairwise *t*-test with Benjamini-Hocberg FDR correction), and fold change >3.0 in any of the groups FA or A1-A4 when compared to the HF control. This resulted in 106 entities that were further reduced to 50 metabolites after cleaning the data from fragments and dimers of the molecular ions, from entities having high (>20%) RSD values in more than 3 out of 6 conditions, peaks with bad quality, or entities with clearly integration mistakes. The final set of metabolites was identified based on the auto MS/MS data or within targeted MS/MS analysis for extracted ions. The fragmentations were compared against The METLIN Metabolite Database (http://metlin.scripps.edu/), Human Metabolome Database (HMDB, http://www.hmdb.ca/), or earlier published fragmentation patterns, and verified with commercial standards when available. Bar chart results are expressed as means ± SEM of peak area abundances (GraphPad Prism Version 5.03). K-means clustering analyses were used for further visualization of the final set of 50 metabolites by using Multi Experiment Viewer, MeV (version 4.8.1) (http://www.tm4.org/). The raw data was imported to MeV and the data were normalized in row-wise manner aiming at comparing the metabolite intensities between different samples (Value = [(Value) – Mean(Row)]/[Standard deviation(Row)]). The normalized data were then clustered into three K-means clusters according to metabolites using a Pearson correlation as a distance metric method with maximum of 50 iterations.

## Results and discussion

**Figure 1:**
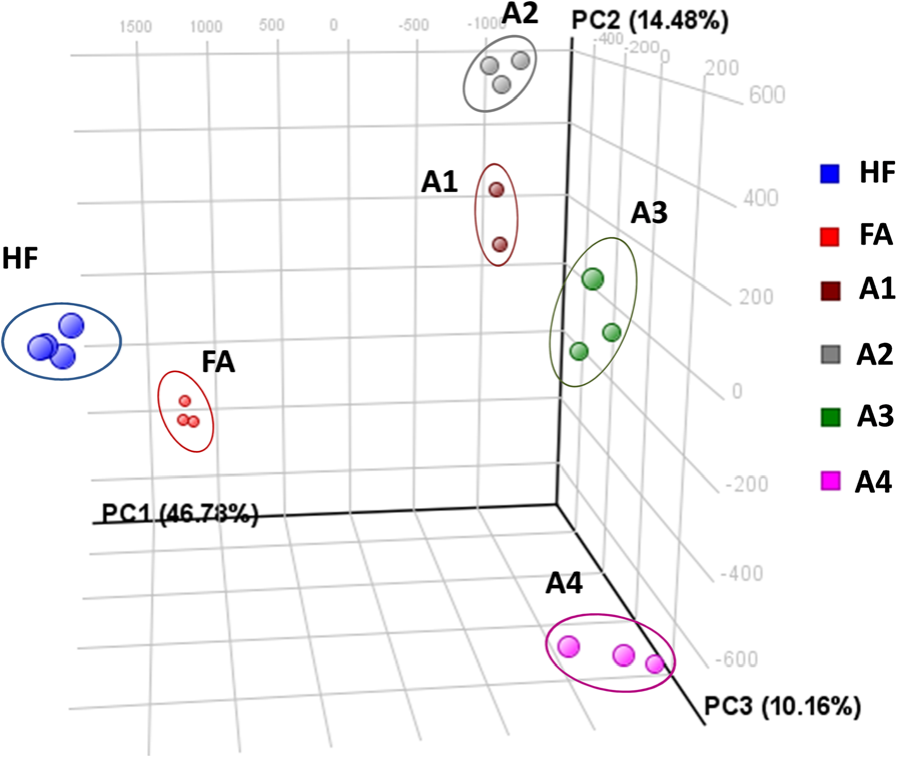
**Principal component analysis (PCA) of the urinary metabolite features resulting from HILIC chromatography combined with ESI(-)qTOF-MS non-targeted metabolite profiling.** Diet induced obese mice were fed with high-fat control diet (HF), or with HF diet containing either free commercial ferulic acid (FA) or one of the four differently processed aleurone preparations (A1-A4).

**Figure 2:**
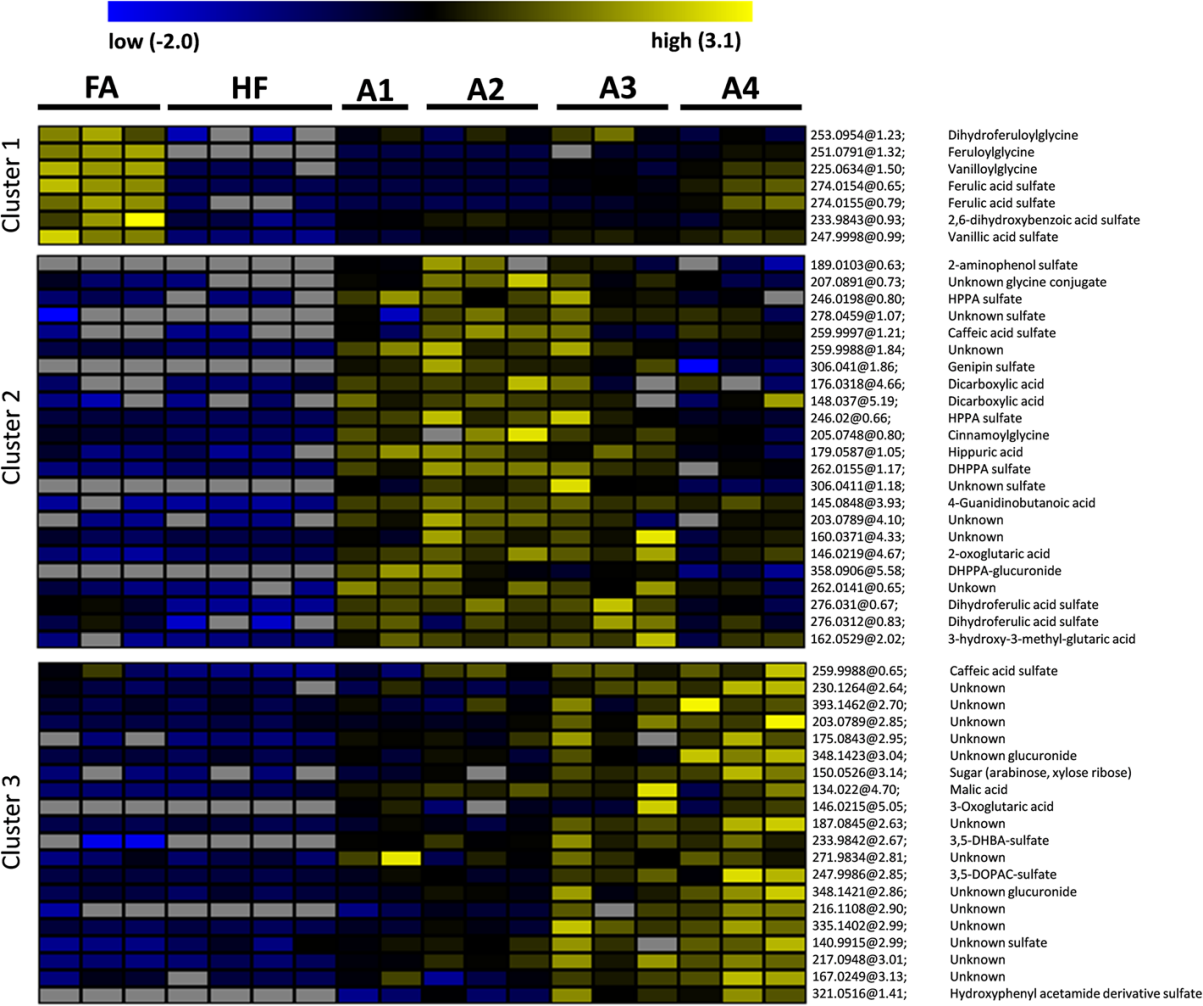
**K-means clustering analysis on the 50 significantly increasing (q < 0.05) urinary metabolite features after adding FA or one of the aleurone preparations (A1-A4) into the diet when compared to HF control diet.** The signal abundances were row-wise normalized and classified into three different clusters. The color-coding scale indicates the abundance within each metabolite: blue: low abundance, yellow: high abundance, gray: not detected. Each replicate is a pool of 2–3 mice on the same diet group.

Principal component analysis of the dataset containing ANOVA-filtered metabolite entities (q < 0.05) showed clear clustering of the biological sample replicates close to each other with clear separation between the dietary groups (Figure 1). The principal component 1 (46.78%) explains the largest variation in the plot referring mainly to the difference caused by the addition of the processed aleurone preparations to the diet. We further narrowed the dataset to only focus on the highly abundant metabolites amenable for MS/MS structural investigation that were significantly increased (q < 0.05, fold change >3) after adding FA or one of the aleurone preparations into the diet when compared to HF control diet. This resulted in 50 metabolites (Table 1). These metabolites were classified into three different clusters according to the occurrence of the metabolite signal in the different groups by using K-means cluster analysis (Figure 2).

**Table 1:** Complete list of metabolites

**MS *m/z*** **(-)**	**Retention time**	**MS/MS *m/z *** **ES(-)**	**Tentative structural assignment**	**Molecular formula**	**MM**	**Cluster**	**Ref./Metlin ID**
188.0023	0.63	188.0023 [M-H]^-^ 10ev: 188.0025 [M-H]^-^ (100), 79.9574 [SO3]^-^ (74), 108.0456 [M-H-SO3]^-^ (59), 80.9657 (3)	*2-Aminophenol sulfate*	C6H7NO4S	189.0103	Cluster 2	[15]
258.9908	0.65	258.9908 [M-H]^-^ 20ev: 179.0351 [M-SO3]^-^ (100), 135.0451 [M-SO3-COO]^-^ (95), 96.9609 [HSO4]^-^ (5), 135.0941 (2)	*Caffeic acid sulfate*	C9H8O7S	260.00	Cluster 3	[16]
261.0061	0.65	261.0061 [M-H]^-^	*Unknown M- [261.0061] rt 0.65*		262.0141	Cluster 2	N.I.
273.0074	0.65	273.0075 [M-H]^-^ 10ev: 193.0508 [M-H-SO_3_]^-^ (100), 273.0076 [M-H]^-^ (33), 178.0266 [M-H-SO3-CH3]^-^ (7), 149.0604 [M-H-SO3-COO]^-^ (6), 96.9598 [HSO4]^-^ (5), 193.1079 (5), 134.0365 (5), 229.0171 (3), 79.9571 [SO3]^-^ (1)	*Ferulic acid sulfate (3- or 4-sulfate) - FA sulfate*	C10H10O7S	274.0154	Cluster 1	[16]
245.012	0.66	245.0144 [M-H]^-^ 10ev: 245.0119 [M-H]^-^ (100), 165.0547 [M-H-SO3]^-^ (34), 121.065 [M-H-SO3-COO]^-^ (10), 245.0757 (4), 79.957 [SO3]^-^ (2)	*3-or 4-Hydroxyphenylpropionic acid sulfate - HPPA-sulfate*	C9H10O6S	246.02	Cluster 2	MID 4152
275.023	0.67	275.0237 [M-H]^-^ 20ev: 195.0664 [M-H-SO3]^-^ (100), 59.0138 (54), 79.9582 [SO3]- (31), 136.0526 (25), 275.024 [M-H]- (14), 151.0755 [M-H-SO3-COO]^-^ (7)	*Dihydroferulic acid sulfate*	C10H12O7S	276.031	Cluster 2	[16]
206.0811	0.73	206.0838 [M-H]^-^ 10ev: 74.0251 [Glycine-H]^-^ (100), 206.0817 [M-H]^-^ (36), 162.0925 [M-H-COO]^-^ (9), 131.0502 [M-glycine]^-^ (3), 126.0264 (3)	*Unknown glycine conjugate M- [206.0838] rt 0.73*	C11H13NO3	207.0891	Cluster 2	N.I.
273.0075	0.79	273.0075 [M-H]^-^10ev: 193.0508 [M-H-SO_3_] (100), 273.0075 [M-H]^-^ (42), 96.9608 [HSO_4_]^-^ (9), 149.0603 [M-H-SO3-COO] (9), 178.0272 (8)	*Ferulic acid sulfate (3- or 4-sulfate) - FA sulfate*	C10H10O7S	274.0155	Cluster 1	[16]
204.0668	0.80	204.0667 [M-H]^-^ 10ev: 160.0768 [M-H-COO]^-^ (100), 130.0663 [M-H-C2H4NO2]^-^ (37), 204.0667 [M-H]^-^ (29), 103.0552 [M-C3H3NO3]^-^ (27), 117.0707 (8), 132.0822 (6), 82.0288 (4)	*Cinnamoylglycine*	C11H11NO3	205.0748	Cluster 2	MID 34534
245.0118	0.80	245.0144 [M-H]^-^ 10ev: 245.0119 [M-H]^-^ (100), 165.0547 [M-SO3]- (30), 121.065 [M-H-SO3-COO]- (11), 74.0241 (8), 79.957 [SO3]- (2), 201.1035 [M-H-COO]^-^ (2)	*3-or 4-Hydroxyphenylpropionic acid sulfate - HPPA-sulfate*	C9H10O6S	246.0198	Cluster 2	MID 4152
275.0232	0.83	275.024 [M-H]^-^ 10ev: 275.024 [M-H]^-^ (100), 195.066 [M-H-SO3]^-^ (41), 193.051 (12), 79.9582 [SO3]^-^ (8), 59.0138 (2), 136.054 (2), 80.9653 (2), 151.076 [M-H-SO3-COO]^-^ (1), 149.061 (1), 123.045 (1)	*Dihydroferulic acid sulfate*	C10H12O7S	276.0312	Cluster 2	[16]
232.9763	0.93	232.976 [M-H]^-^ 20ev: 153.0193 [M-H-SO3]^-^ (100), 109.0294 (90.24), 135.0446 (14.23), 96.9596 [HSO4]^-^ (5.32), 123.0434 (2.33), 61.9895 (2.23), 232.975 [M-H]^-^ (2.12)	*2,6-Dihydroxybenzoic acid sulfate - 2,6-DHBA sulfate*	C7H6O7S	233.9843	Cluster 1	[17]
246.9918	0.99	246.9918 [M-H]^-^ 10ev: 167.0354 [M-H-SO_3_]^-^ (100), 152.0118 (88), 123.0118 (41), 108.0218 (25), 96.906 [HSO4]^-^ (9), 152.0639 (6), 79.9581 [SO3]^-^ (4)	*Vanillic acid sulfate*	C8H8O7S	247.9998	Cluster 1	Manual
178.0507	1.05	178.0521 [M-H]^-^ 10ev: 134.0612 [M-H-COO]^-^ (100), 178.0511 [M-H]^-^ (85.7), 77.0401 [M-H-C3H3NO3]^-^ (41.4), 56.0147 (6.0)	*Hippuric acid*	C9H9NO3	179.0587	Cluster 2	MID 1301
277.0379	1.07	277.0379 [M-H]- 20ev: 197.0811 [M-H-SO3]- (100), 182.0574 [M-H-SO3-CH3]- (59), 79.9577 [SO3]^-^ (29), 197.0453 (20), 125.0608 [C7H9O2] (20), 277.0371 [M-H]^-^ (18), 122.036 (16),	*Unknown sulfonated metabolite M- [277.0379] rt 1.071*		278.0459	Cluster 2	N.I.
261.0075	1.17	261.0081 [M-H]^-^ 20ev: 137.061 (100), 181.0504 [M-SO3]^-^ (14), 261.0072 [M-H]^-^ (13), 95.0504 (8), 79.9568 [SO3]^-^ (3)	*3,4- or 3,5-Dihydroxyphenylpropionic acid sulfate - DHPPA-sulfate*	C9H10O7S	262.0155	Cluster 2	Unconjugated std.
305.0331	1.18	305.0343 [M-H]^-^ 20ev: 225.0783 [M-H-SO3]^-^ (100), 59.0147 (90), 79.9572 [SO3]^-^ (48), 166.0626 (36), 80.9651 (13), 149.0605 (12), 165.0567 (12), 153.0552 (8), 305.0386 [M-H]^-^ (8)	*Unknown sulfated metabolite M- [305.0331]- rt 1.18*	C11H14O8S	306.0411	Cluster 2	N.I.
258.9917	1.22	258.9926 [M-H]- 20ev: 179.0352 [M-H-SO3]^-^ (100), 135.045 [M-SO3-COO]^-^ (9), 91.0553 [C7H7]- (5), 258.9926 [M-H]- (4), 93.0357 [C6H5O]- (3)	*3- or 4-Caffeic acid sulfate*	C9H8O7S	259.9997	Cluster 2	[16]
252.0874	1.23	252.0896 [M-H]^-^10ev: 74.0255 [C2H4NO2]^-^ (100), 252.0889 [M-H]^-^ (74), 193.0769 [C10H9O4]^-^ (10), 177.0554 [M-H-glycine]^-^ (8)	*Dihydroferuloylglycine*	C12H15NO5	253.0954	Cluster 1	Manual
250.0711	1.32	250.0711 [M-H]^-^ 10ev: 100.0035 (100), 149.0608 (91), 250.072 [M-H]^-^ (80), 206.0828 [M-H-COO]^-^ (68), 134.0377 (68), 163.0637 (29), 191.9576 (22), 175.0545 (20), 177.0545 (19)	*Feruloylglycine*	C12H13NO5	251.0791	Cluster 1	[16]
320.0436	1.42	320.0436 [M-H]^-^ 20ev: 230.0134 [M-H-C3H6O3]^-^ (100), 150.0557 [M-H-C3H6O3-SO3]^-^ (29), 320.0453 [M-H]^-^ (14), 79.9575 [SO3]^-^ (11), 108.0444 [C6H6NO]^-^ (9), 148.0406 (9), 80.9662 (6)	*Sulfonated Hydroxyphenylacetamide + C3H6O3*	C11H15NO8S	321.0516	Cluster 3	HPAA std.
224.0554	1.50	224.0566 [M-H]^-^ 10ev: 100.0042 [C_3_H_2_NO_3_]^-^ (100), 224.0566 [M-H]^-^ (38), 123.0449 [C7H7O2]^-^ (31), 74.0230 (12), 180.0646 [M-H-COO]^-^ (9), 165.0402 [M-H-COO-CH3]^-^ (7)	*Vanilloylglycine*	C10H11NO5	225.0634	Cluster 1	[18]
258.9908	1.84	258.9938 [M-H]^-^ 10ev: 215.0026 [M-H-COO]^-^ (100), 135.045 [M-H-COO-SO3]^-^ (22), 240.9815 [M-H-H2O]^-^ (7), 161.0245 (6), 258.9934 [M-H]^-^ (0.4)	*Unknown M- [258.9938] rt 1.84*	C9H8O7S	259.9988	Cluster 2	N.I.
305.033	1.86	305.0354 [M-H]^-^ 20ev: 123.0452 (100), 101.0244 (83), 163.0763 (62), 305.0354 [M-H]^-^ (61), 207.0688 (8) [M-H2O-sulfate], 79.9573 [SO3]^-^ (7), 96.9606 [HSO4]^-^ (6), 287.0189 [M-H2O]^-^ (2)	*Genipin sulfate*	C11H14O8S	306.041	Cluster 2	[19]
161.0449	2.02	161.0450 [M-H]^-^ 10ev: 57.0348 [C3H5O]^-^ (100), 99.0453 [C5H7O2]^-^ (80), 101.0241 [M-H-C2H4O2]^-^ (40), 161.045 [M-H]^-^ (29), 59.0144 (11)	*3-hydroxy-3-methyl-glutaricacid - HMGA*	C6H10O5	162.0529	Cluster 2	MID 3793
186.0765	2.63	186.0765 [M-H]- 10ev: 114.0927 [M-H-C2CO3]^-^ (100), 142.0866 [M-H-COO]^-^ (54), 186.0779 [M-H]^-^ (52), 58.03 [C2H4NO]^-^ (39), 100.0757(37), 68.0508 [C4H6N]^-^ (18)	*Unknown N-containing metabolite M*^*-*^*[186.0765] rt 2.63*	C8H13NO4	187.0845	Cluster 3	N.I.
229.1184	2.64	229.1184 [M-H]^-^ 10ev: 229.1179 [M-H]^-^ (100), 187.1077 [M-H-C2H2O]^-^ (86), 58.0301 [C2H4NO] (13), 145.0978 [C6H13N2O2]^-^ (12), 143.1194(12)	*Unknown N-containing metabolite M*^*-*^*[229.118] rt 2.64*	C10H18N2O4	230.1264	Cluster 3	N.I.
232.9762	2.67	232.9779 [M-H]^-^ 20ev: 153.0195 [M-H-SO3]^-^ (100), 109.0297 [M-H-SO3-COO]^-^ (29), 65.0405 (21), 67.0198 (12), 232.9776 [M-H]^-^ (4)	*3,5-dihydroxybenzoic acid sulfate - 3,5-DHBA sulfate*	C7H6O7S	233.9842	Cluster 3	Unconjugated std./[17]
392.1382	2.70	392.1382 [M-H]^-^ 20ev: 124.0071 (100), 348.1493 [M-H-COO]^-^ (54), 146.8045 (6), 79.9583 [SO3]^-^ (3), 149.9861 (3)	*Unknown metabolite M- [392.1382] rt 2.70*	C16H27NO8S?	393.1462	Cluster 3	N.I.
270.9754	2.81	270.9754 [M-H]^-^ 20ev: 96.9602 [HSO4]^-^ (100), 97.0011 (2), 79.9562 [SO3]^-^ (2), 112.987 (1), 270.9775 (1)	*Unknown sulfate containing metabolite M- [270.9754] rt 2.8*	C6H8O10S?	271.9834	Cluster 2	N.I.
202.0709	2.85	202.0709 [M-H]^-^ 10ev: 202.0721 (M-H]^-^ (100), 88.0405 [C3H6NO2] (80), 158.0818 [M-H-COO]^-^ (56), 86.0615 (54), 87.0451 [C4H8NO] (43), 114.0194 (26), 184.0607 [M-H-H2O]^-^ (24), 140.0706 [C7H10NO2]^-^ (14), 70.6966 (7), 132.0306 (6)	*Unknown N-containing metabolite M- [202.0709] rt 2.85*	C8H13NO5	203.0789	Cluster 3	N.I.
246.9906	2.85	246.9931 [M-H]- 10ev: 167.0356 [M-SO3]^-^ (100), 246.9931 [M-H]^-^ (44), 123.0458 [M-SO3-COO]^-^ (13), 96.9607 [HSO4]- (8), 152.0123 [M-SO3-COO-CH3]^-^ (8), 203.0024 (7), 167.0923 (6), 79.9581 [SO3]^-^ (3)	*3,5-Dihydroxyphenyl acetic acid sulfate - 3,5-DOPAC sulfate*	C8H8O7S	248.00	Cluster 3	Unconjugated std.
347.1341	2.86	347.1370 [M-H]^-^	*Unknown glucuronidated metabolite M- [347.1370-glucuronide -- > 171.1031]*	C15H24O9	348.1421	Cluster 3	N.I.
215.1028	2.91	215.1028 [M-H]^-^ 20ev: 173.0925 [M-H-C2H2O]^-^ (100), 58.03 [C2H4NO]^-^ (51), 44.0145 (42), 143.1179 (24), 129.1032 (11), 126.0921 (6), 172.1036 (3)	*Unknown N-containign metabolite M*^*-*^*[215.103] rt 2.91*	C9H16N2O4	216.1108	Cluster 3	N.I.
174.0763	2.95	174.0763 [M-H]^-^ 10ev: 74.025 [glycine]^-^ (100), 174.0766 [M-H]^-^ (42), 100.0758 [M-H-C2H2O3]^-^ (12), 128.0703 (11), 112.0374 (10), 130.085 (9), 132.067 (5), 59.0132 (4)	*Unknown N-containign metabolite M- [174.0763]*	C7H13NO4	175.0843	Cluster 3	N.I.
334.1322	2.99	334.1322 [M-H]^-^	*Unknown metabolite M- [334.132] rt 2.99*	C14H25NO6S?	335.1402	Cluster 3	N.I.
139.9835	2.99	139.9859 [M-H]- 10ev: 95.9344 [M-H-COO]^-^ (100), 139.9842 [M-H]- (94), 76.0225 [C2H6NS]^-^ (5), 96.9419 (3), 95.9763 (2), 140.0336 (2)	*Unknown sulfate containing metabolite M- [139.9859] rt 2.99*	?	140.9915	Cluster 3	N.I.
216.0868	3.01	216.0868 [M-H]^-^ 10ev: 128.0342 (100), 172.0965 [M-H-COO]^-^ (90), 216.0872 [M-H]^-^ (87), 198.0769 [M-H-H2O]^-^ (31), 102.0566 (28), 128.1066 (26), 87.0459 (21), 86.0603 (20), 98.06 (12), 154.0854 (12)	*Unknown N-containing metabolite M- [216.0868] rt 3.01*	C9H15NO5	217.0948	Cluster 3	N.I.
347.1343	3.04	347.1370 [M-H]^-^ 10ev: 113.0247 [fragment of glucuronide] (100), 171.1031 [M-glucuronide]^-^ (85), 347.1361 [M-H]^-^ (30), 85.0294 [fragment of glucuronide] (25), 175.0247 (23), 59.0146 (20), 95.0132 (13)	*Unknown glucuronidated metabolite [m/z 171.1023 + glucuronide]*	C15H24O9	348.1423	Cluster 3	N.I.
166.0169	3.13	166.0169 [M-H]- 20ev: 79.9578 [SO3]^-^ (100), 166.0167 [M-H]^-^ (28), 106.9804 (25), 100.0411 (13), 80.9651 (9), 58.0295 (5)	*Unknown M- [166.017] rt 3.13*	C4H9NO4S	167.0249	Cluster 3	N.I.
149.0446	3.14	149.0466 [M-H]^-^	*Tentative sugar either arabinose, xylose, or ribose*	C5H10O5	150.0526	Cluster 3	N.I.
144.0768	3.94	144.0790 [M-H]^-^ 10ev: 102.0564 [M-H-CH2N2]^-^ (100), 83.0619 (12), 84.0453 [M-H-COO-NH3]^-^ (7), 58.0415 [C3H6O]^-^ (6), 144.0778 [M-H]^-^ (5), 102.099 (2)	*4-Guanidinobutanoic acid*	C5H11N3O2	145.0848	Cluster 2	MID 4155
202.0709	4.10	202.0733 [M-H]^-^ 10ev^:^ 140.0716 (100), 158.0824 [M-H-COO]^-^ (99), 202.0724 [M-H]^-^ (93), 58.0297 [C2H4NO]^-^ (50), 100.0401 [C4H6NO2]^-^ (49), 116.069 (33), 69.0364 (31), 98.0612 (31), 110.0603 (28), 75.7348 (24), 345.1717 (12), 176.0227 (12)	*Unknown N-containing metabolite M- [202.071] rt 4.10*	C8H13NO5	203.0789	Cluster 2	N.I.
159.0291	4.33	159.0291 [M-H]^-^	*Unknown M- [159.0291] rt 4.33*	C6H8O5	160.0371	Cluster 2	N.I.
175.0238	4.66	175.0238 [M-H]^-^ 10ev: 157.0134 [M-H-H2O]^-^ (100), 87.0453 [C4H7O2]^-^ (28), 175.0249 [M-H]^-^ (20), 132.0666 [M-H-CH3CO]- (16), 69.0346 (15)	*Unknown tentative dicarboxylic acid*	C6H8O6	176.0318	Cluster 2	N.I.
145.0139	4.67	145.0139 [M-H]^-^ 10ev: 101.0246 [M-H-COO]^-^ (100), 57.0348 [C3H5O]^-^ (25), 145.0135 [M-H]^-^ (16), 73.0296 [C3H5O2]^-^ (5)	*2-Oxoglutaric acid*	C5H6O5	146.0219	Cluster 2	MID 119
133.014	4.70	133.014 [M-H]^-^ 10ev: 115.0036 [M-H-H2O]^-^ (100), 71.014 [C3H3O2]^-^ (60), 133.014 [M-H]^-^ (38), 72.993 [C2HO3]^-^ (16), 89.0246 [M-H-COO]^-^ (9), 87.0077 (2), 44.9976 (2)	*Malic acid*	C4H6O5	134.022	Cluster 3	MID 118
145.0135	5.05	145.0135 [M-H]^-^ 10ev: 101.024 [M-H-COO]^-^ (100), 57.0352 [C3H5O]^-^ (97), 145.0116 [M-H]^-^ (26), 82.028 (6), 127.0225 [M-H-H2O]^-^ (6)	*3-Oxoglutaric acid*	C5H6O5	146.0215	Cluster 3	Manual
147.029	5.19	147.029 [M-H]^-^	*Undientified M*^*-*^*[147.029]*	C5H8O5	148.037	Cluster 2	
357.0826	5.59	357.0855 [M-H]^-^ 10ev: 357.0843 [M-H]^-^ (100), 113.0237 [fragment of glucuronide]^-^ (58), 181.0518 [M-H-glucuronide]^-^ (13), 137.0596 [M-H-glucuronide-COO]^-^ (10), 175.0238 (10), 59.0138 (10), 85.0287 [fragment of glucuronide]^-^ (6)	*Glucuronidated 3,4- or 3,5-Dihydroxyphenylpropionic acid, DHPPA-glucuronide*	C15H18O10	358.0906	Cluster 2	[15]

### Majority of free ferulic acid was excreted as sulfate or glycine conjugates

**Figure 3:**
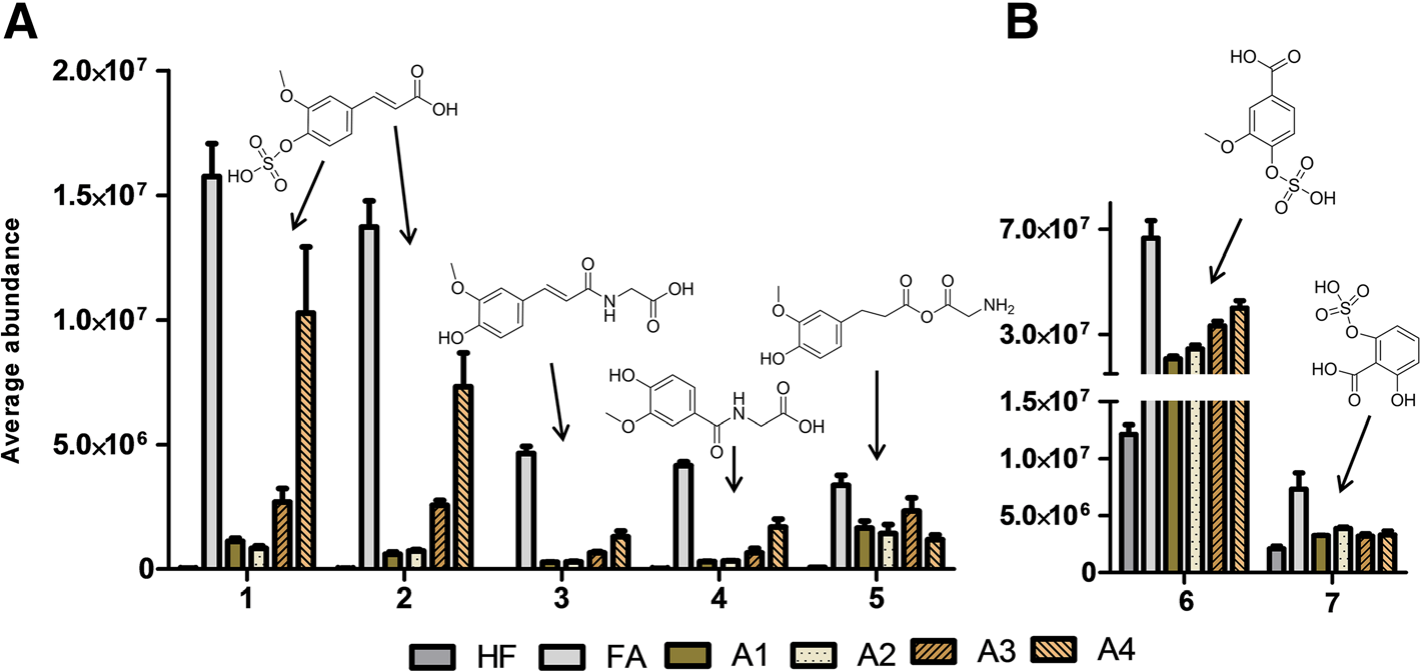
**The urinary metabolites found in the cluster 1. A**: Compounds that were highly increased in urine of mice fed either free commercial ferulic acid (FA) or one of the four differently processed aleurone preparations (A1-A4) but were not present in the in the HF control group. **B**: compounds that were increased duoe to the test diets, but had also high basal abundance in the HF control group. 1. 3- or 4-ferulic acid sulfate, 2. 3- or 4-ferulic acid sulfate, 3. feruloylglycine, 4. vanilloylglycine, 5. dihydroferuloylglycine, 6. vanillic acid sulfate, and 7. 2,6-DHBA sulfate.

The cluster 1 includes metabolites that were highly increased after adding FA into the diet for 8 weeks (fold change to HF control was between 421 and 4.5-fold) (Figure 2). By comparing the MS/MS fragmentation data with those given in public databases (METLIN, HMDB) or with the unconjugated standard compounds these metabolite signals in the cluster 1 were identified to be two different isomers of ferulic acid sulfates (*m/z* 273.0075), unidentified *m/z* 246.9918, glycine conjugate of ferulic acid (feruloylglycine, *m/z* 250.0711), glycine conjugate of vanillic acid (vanilloylglycine *m/z* 224.0554), dihydroferuloylglycine *(m/z* 252.0874), and 2,6- dihydroxybenzoic acid sulfate (*m/z* 232.9763, (Figure 3, Table 1). Majority of the metabolites in the cluster 1 seem to be well-known endogenous metabolites of rapidly absorbed FA. FA sulfate has been identified as major dietary FA metabolite recovered in urine in rats [20-23]. Previous *in vivo* studies have shown that free dietary FA is rapidly absorbed in the upper parts of the intestine and conjugated mainly in the liver either into its phase II metabolites, mainly sulfonate or glucuronide conjugates and further excreted in urine [22]. A small proportion of ingested free FA may also be metabolized through beta-oxidation in liver to form vanillic acid, or through phase I metabolism into dihydroferulic acid and different forms of dihydroxybenzoic acids [20,24,25]. Notably, the cluster 1 was particularly rich in glycine conjugated metabolites.

The FA diet contained pure FA added to the food mass and was thus in free form which allows the solubilization and absorption from the upper parts of the intestine. However, this is not the case with more complex biological matrices. In the unprocessed aleurone FA is covalently bound to the arabinoxylan and the complex structure of the aleurone does not allow absorption of FA [10]. Therefore, prior to reaching the circulation FA moieties need to be released from the surrounding tissue matrix either by the intestinal mucosal enzymes or by the intestinal bacterial enzymes, in order to be bioavailable for the circulation and human tissues thereafter, to exert any potential bioactivity on cellular metabolism [8,9]. Therefore, attempts have been made to improve the bioavailability of FA by different bioprocessing techniques, such as fermentation or enzymatic treatments of cereal grain, bran or aleurone matrices [7,12-14]. Interestingly, the A4 diet (xylanase and A-type feruloyl esterase treatment) produced a similar excretion profile of the main liver phase II metabolites of FA, especially of FA sulfates, as the diet containing free FA but in lower abundance (Figure 3). Furthermore, the urine of the mice in A4 group displayed clearly different excretion pattern of FA metabolites than the other aleurone groups A1-A3, suggesting that the bioavailability of FA in A4 group was, indeed, improved. The diet containing aleurone treated only with xylanase (A3) also generated increased free FA and FA linked to oligosaccharides (42% of total FA), and there was a tendency of increased urinary excretion of the major FA metabolites found in the cluster 1 (Figure 3A). The resemblance of FA related metabolite profile (cluster 1) between A4 and FA groups is not surprising since the combination of xylanase and feruloylesterase treatment resulted in an increased amount of FA moieties released in free form or as conjugated to oligosaccharides (85% of total FA) in the A4 aleurone fraction and the A4 diet [14]. Previous *in vitro* studies have also suggested that enzymatic treatments by xylanase combined or not with feruloylesterase could improve the bioavailability of FA from the aleurone layer [13]. Here we show for the first time that an enzymatic modification applied to aleurone layer can affect also *in vivo* metabolism of FA, the main phenolic acid present in the aleurone.

The most intense metabolite signal in the cluster 1 was the *m/z* of 246.9918. The MS/MS analyses created fragments that have been reported for both dihydroxyphenylacetic acid sulfate (DOPAC sulfate) and vanillic acid sulfate [26,27]. The further analysis with commercial standard compounds (vanillic acid, DOPAC) suggested that the compound is rather vanillic acid sulfate than DOPAC sulfate according to their fragmentation patterns. Even though these two compounds share similar fragments (167.0352 [M-H-sulfate]^-^, 123.0453), it is most likely that the fragments of 108.0218 and 152.0115 distinguish between these two compounds (Additional file 1).

Tentatively identified DHBA-sulfate of the cluster 1 metabolites was also detected in high abundance in the HF control group, suggesting that there are also other dietary factors that might influence the high basal excretion of this metabolite. The MS/MS fragmentation of the DHBA sulfate (*m/z* 232.9763) created a fragment ion of *m/z* 135.0195, which has been suggested to derive only from the 2,6-configured DHBA (γ-resorcylic acid) [17]. This metabolite has been reported as a significant plasma marker of fibre intake in a 5-weeks human intervention, and has been suggested to be originated from the alkylresorcinols after microbial metabolization [17]. However, our results suggest that the source of 2,6-DHBA might be the phenolic acids, naturally present within the diet as this form of DHBA was mainly excreted in urine after addition of FA into the diet. The colonic bacteria are capable of reconfiguring the phenolic hydroxyl substitutions, and it is possible that part of the free FA can escape absorption and end up to the distant parts of the intestine for microbial metabolism. Furthermore, the 3,5-DHBA having different hydroxyl group configuration is better described as the alkylresorcinol intake biomarker [28], and it was located into cluster 3 in our study.

### Native and cryoground aleurone increased the excretion of microbial phenolic metabolites

**Figure 4:**
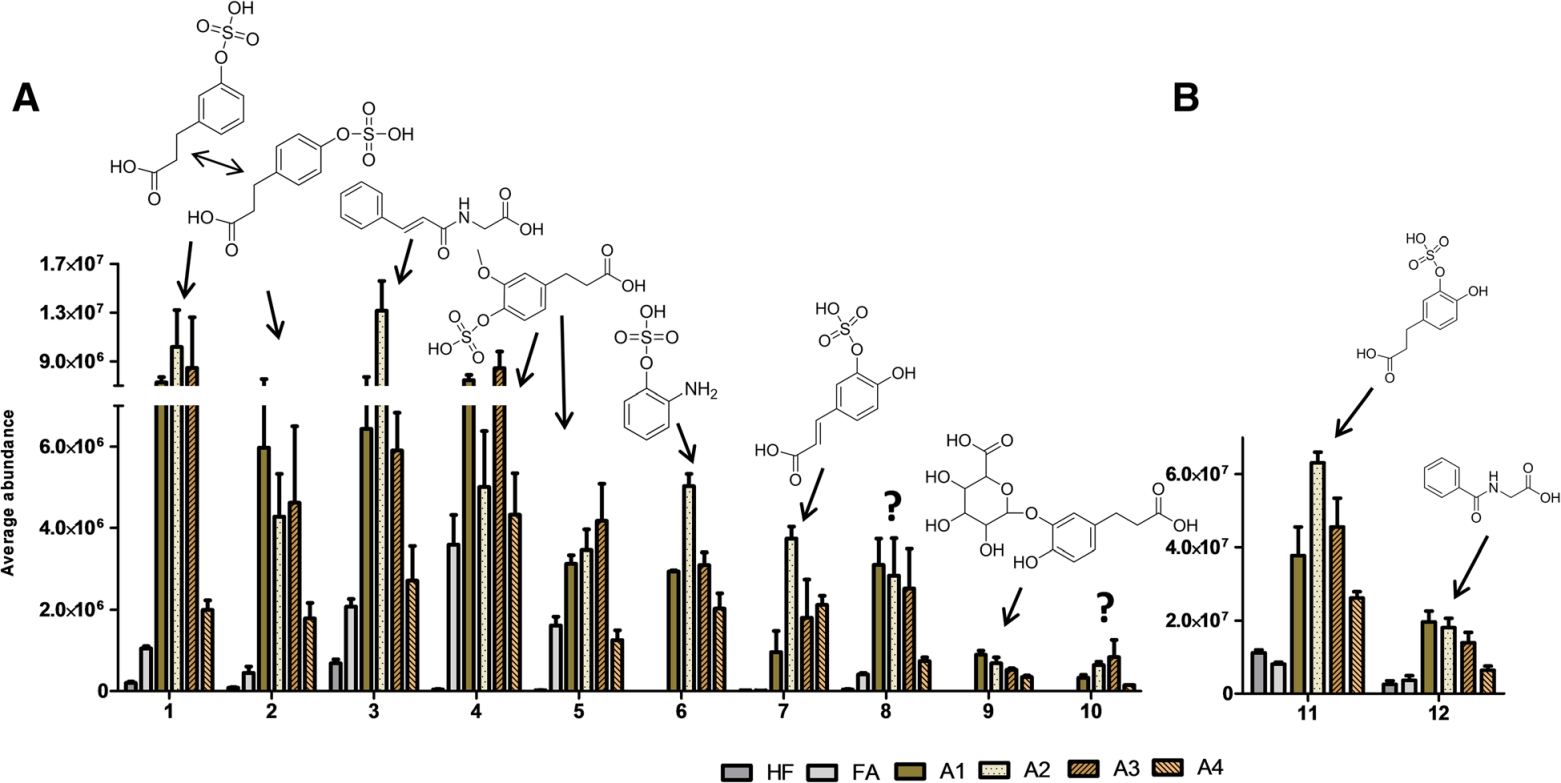
**The urinary metabolites from the cluster 2. A**: 1. 3- or 4-HPPA sulfate, 2. 3- or 4-HPPA sulfate, 3. cinnamoylglycine, 4. 3 or 4-dihydroferulic acid sulfate, 5. 3- or 4-dihydroferulic acid sulfate, 6. aminophenolsulfate, 7. 3 or 4-caffeic acid sulfate, 8. *m/z* 258.99, 9. glucuronidated DHPPA, 10. tentative dihydrosinapic acid sulfate. **B**: 11. DHPPA sulfate and 12. hippuric acid (note the different scale).

The majority of the urinary metabolites found in cluster 2 are metabolites that have highest abundance in urine of the groups receiving either the native (A1) or ultra-fine ground (A2) aleurone preparation (Figure 2). Interestingly, several of these metabolite features seem to be increased especially in the A2 group (Figures 2 and 4). Furthermore, these metabolites are excreted with a higher abundance in the dietary groups receiving native, cryoground, or xylanase processed aleurone (A1-A3) than in the group receiving xylanase and feruloylesterase processed aleurone (A4) (Figure 4). Among others, the metabolite signals in the cluster 2 included tentatively identified negative ions of *m/z* 261.0075 (dihydroxyphenylpropionic acid sulfate; DHPPA sulfate), *m/z* 178.0507 (hippuric acid), *m/z* 245.0116 in two isomers (hydroxyphenylpropionic acid sulfate; HPPA sulfate), *m/z* 204.0668 (cinnamoylglycine), *m/z* 275.0232 in two isomers (dihydroferulic acid sulfate), *m/z* 188.0023 (aminophenol sulfate), *m/z* 258.9908 (caffeic acid sulfate), *m/z* 357.0826 (DHPPA glucuronide), and *m/z* 305.0331 (unknown) (Figure 4, Table 1). DHPPA sulfate and hippuric acid were the metabolites with the highest signal abundance in this cluster (Figure 4B). All of these compounds have been identified in human studies and in rodent models as microbial-co-metabolites of phenolic compounds [28-31].

**Figure 5:**
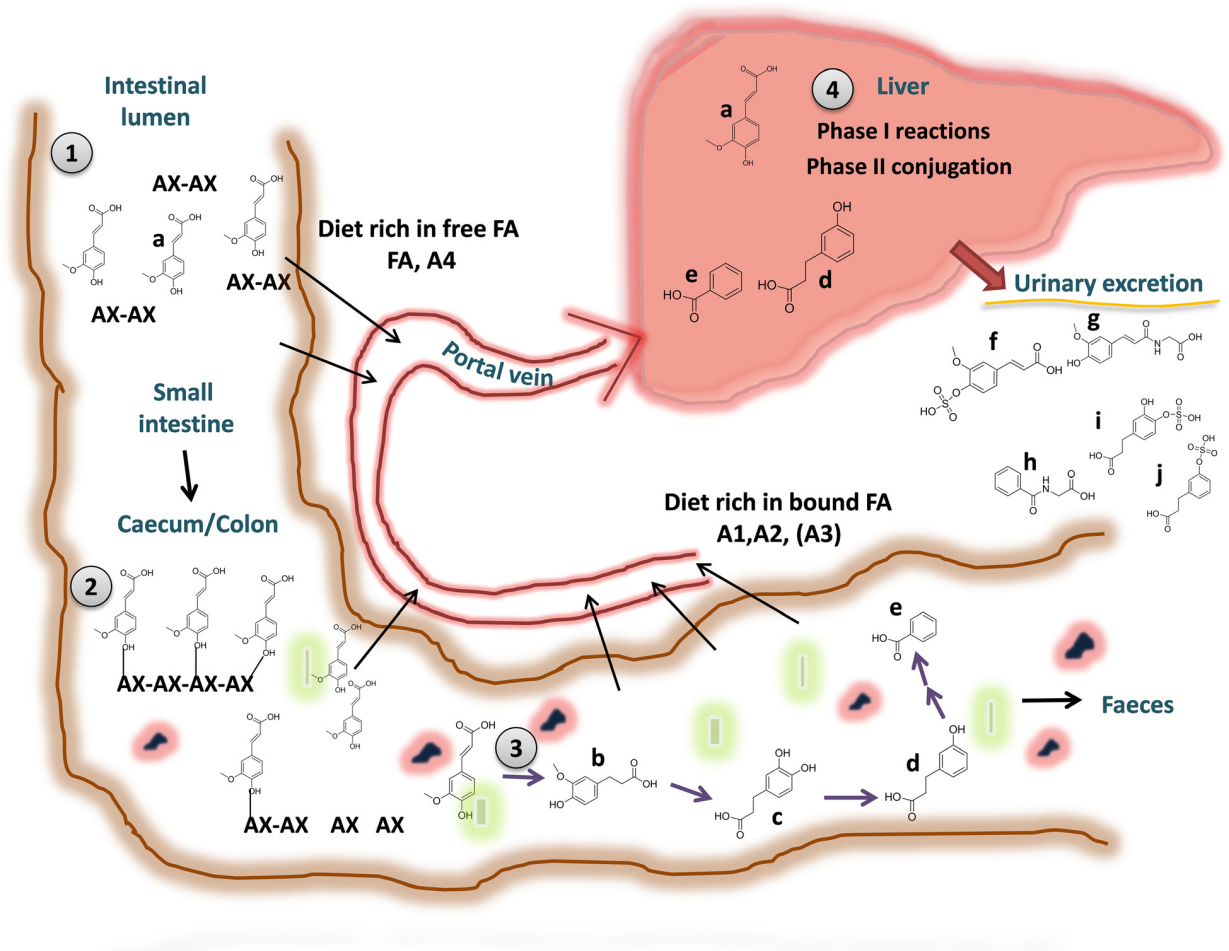
**Schematic presentation on the effect of aleurone structure on ferulic acid absorption from the different diets.** 1. Free FA (a) is readily absorbed to the portal vein from the stomach and upper parts of the intestine. In the diet A4 majority of FA is in free form due to xylanase and feruloylesterase pretreatments. 2. The diet A3 contains xylanase treated aleurone and has a mixture of both free and bound FA and is therefore in brackets. Whereas, in the diets containing intact (A1) or cryo-ground aleurone (A2) majority of FA is tightly bound to the arabinoxylan fibre matrix, and bioavailability of FA is thus dependent on its release by the microbial enzymes within the caecum (mice) and colon. 3. Most of the microbially released FA is further metabolized by the microbial enzymes. The modifications include reduction of FA into dihydroferulic acid (b), further demethylation to form dihydroxyphenylpropionic acid (DHPPA) (c), dehydroxylation into hydroxyphenylpropionic acid (HPPA) (d), and finally after another dehydroxylation step and beta-oxidation to benzoic acid (e). All of these intermediate metabolites can be absorbed to circulation, however, part of them are excreted in faeces. 4. All the absorbed compounds can be further metabolized in liver via phase I xenobiotic metabolism or directly conjugated either with sulfate, glucuronide, or amino acids such as glycine and excreted in urine (phase II). When FA is absorbed from the upper parts of the intestine the main metabolites found in urine are ferulic acid sulfate (f), and feruloylglycine (g). When dietary FA is mainly bound to AX-fibre matrix the main urine metabolites are hippuric acid (h), DHPPA sulfate (i), and HPPA sulfate (j).

Phenolic acids including FA are well-known to face microbial metabolism when they travel along the intestinal tract to the lower parts including caecum (in rodents) and colon. For instance, the double-bond of propenoic acid side chain of FA can be first reduced to form dihydroferulic acid [16], and then further demethylated and dehydroxylated, to form different forms of dihydroxy-, hydroxyl-, or phenylpropionic acids (DHPPA, HPPA or PPA) by the intestinal microbial enzymes (Figure 5) [12,20,30,32]. These metabolites can be further degraded either by the microbial enzymes or by endogenous phase I enzymes into simple benzoic acids, and finally conjugated with glycine in liver to form hippuric acid [33].

The tentatively identified cinnamoylglycine was especially highly increased in the A2 group but also, interestingly, in the FA group (3-fold). However, there are not many studies published about cinnamoylglycine so far. This might be partly because of species specific differences regarding the cinnamoylglycine production, as this metabolite has been detected in urine of mice but not in rats [34].

Notably, in addition to FA, the aleurone layer contains also other but minor phenolic compounds, including sinapic acid and p-coumaric acid [35], which might contribute to the increased excretion of the microbial metabolites of phenolic compounds in the A1-A4 groups when compared to the FA control group. In addition, one of the unidentified metabolites *m/z* 305.0331, which was only detected in the urine of mice receiving aleurone, might indeed be sulfonated dihydrosinapic acid, originating from the sinapic acid (metabolite 10 in Figure 4) (Additional file 1).

Altogether, these results suggest that ferulic acid as well as other phenolic compounds mainly bound to the arabinoxylan in the native and cryo-ground aleurone, are intensively metabolized upon their release within the intestine (caecum, colon) by the action of intestinal microbiota (Figure 5). Therefore, the microbial metabolism could change the fate of the bioactive phytochemicals present in the aleurone similarly as previously shown with different unprocessed and bioprocessed brans [29,36,37]. Whereas, in the case of enzymatically processed A4, the majority of the FA is clearly absorbed already in the upper part of the GI tract and the following metabolism of FA prior to excretion in urine is mainly dependent on the endogenous phase I and phase II metabolism (Figure 5).

### Aleurone addition increased excretion of small dicarboxylic acids

**Figure 6:**
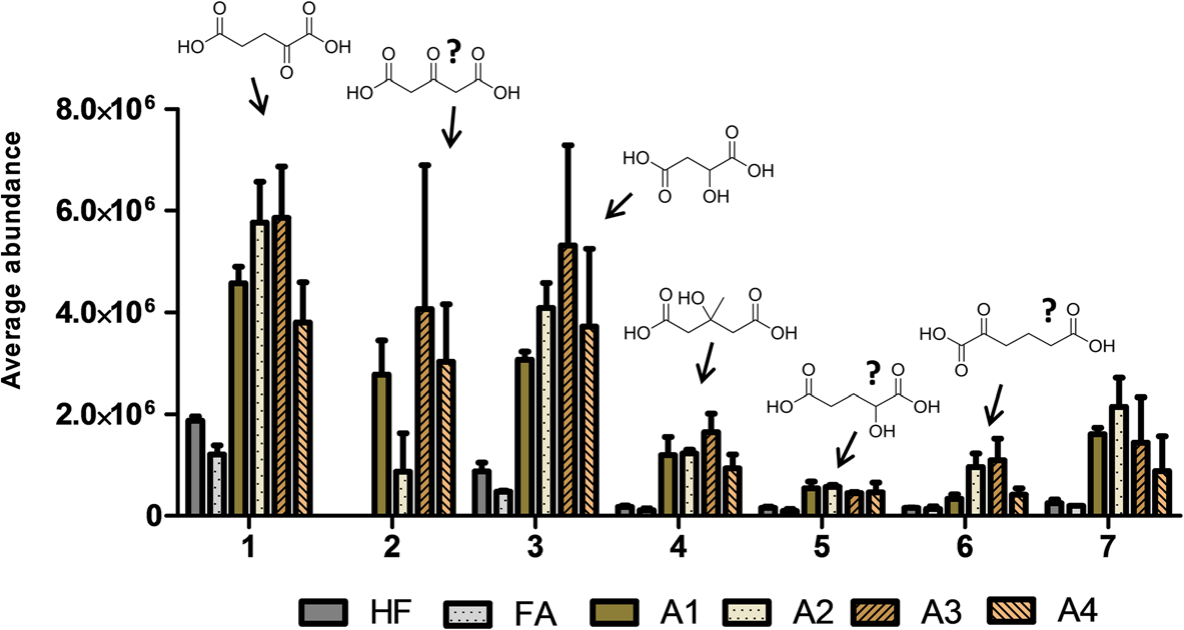
**Excretion of dicarboxylic acids in urine of mice fed diets with free commercial ferulic acid (FA), different aleurone preparations (A1-A4), or with high-fat control diet (HF).** 1. 2-oxoglutaric acid, 2. tentative 3-oxoglutaric acid, 3. malic acid, 4. 3-hydroxy-3-methyl-glutaric acid, 5. *m/z* 147.029, 6*. m/z* 159.0291, 7. *m/z* 175.0238. Metabolite 2 was not present neither in the urine of HF or FA fed mice.

Interestingly, the cluster 2 contained also a set of metabolites sharing similar molecular structure. Two of those were identified as 2-oxoglutaric acid (*m/z* 145.0135) and 3-hydroxy-3-methyl-glutaric acid (HMGA) *m/z* 161.0449 (Figure 6). Additionally, metabolite features of *m/z* 147.029, 159.0291, and 175.0238 shared common fragmentation pattern (Table 1). These urinary metabolites might be derived from the aleurone matrix as several dicarboxylic acids such as malic acid, succinic acid, fumaric acid, and azelaic acid are naturally found in wheat and other grains [38]. Alternatively, they may reflect changes in endogenous metabolism as small organic acids are also naturally present in the human urine [39]. All the metabolites seem to have 4 to 6 carbon backbone, carboxyl groups at the both ends of the molecule, and one or additional oxygens (hydroxyl or acetone) attached to the carbon backbone similarly as several citric acid cycle metabolites. Additionally, two of the dicarboxylic acids were found also in cluster 3, namely malic acid (*m/z* 133.014) and a different isomer of oxoglutaric acid (*m/z* 145.0135) that could be be 3-oxoglutaric acid. All the dicarboxylic acids were increased two to nine-fold in urine of aleurone fed mice but not in urine from the FA fed mice, when compared to the HF control mice. However, very little is known about the dicarboxylic acid group in relation to dietary intake of wheat or grains in general.

### Enzymatically processed aleurone causes increased excretion of several nitrogen-containing metabolites

The majority of the metabolites in the cluster 3 are increased in groups receiving xylanase or xylanase + feruloylesterase processed aleurone (A3 and A4), and especially in the A4 group (Figure 2). The interesting common feature for these metabolites is that they have nitrogen in molecular structure and some of them share similarities in MS/MS fragmentation with the carboxylic or dicarboxylic acids in their main structure (Table 1).

**Figure 7:**
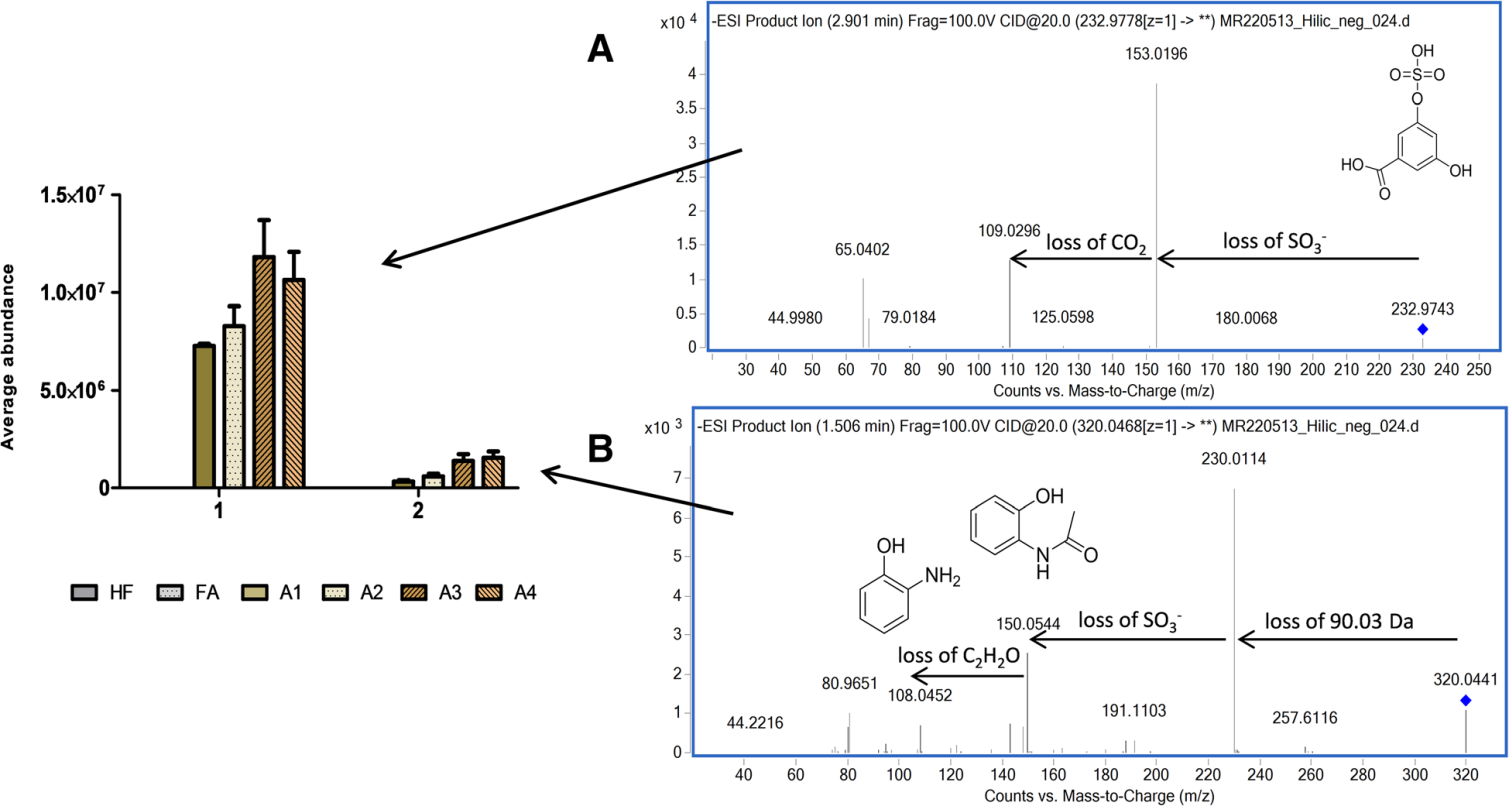
**3,5-DHBA sulfate (A) and a conjugate of hydroxyphenylacetamide sulfate (B) after aleurone addition into the diet.** The ESI(-) MS/MS fragmentation spectra are shown for the both metabolites.

One of the compounds in this cluster (*m/z* 320.0436) formed MS/MS fragment ions of *m/z* 150.0557 and 108.044 (Figure 7B). The fragment ion 150.0557 corresponds to hydroxyphenylacetamide and 108.044 to its basic core structure aminophenol (150.0557 – C2H2O) which both have been reported earlier [15,40]. Additionally, the loss of 79.99 Da indicated that the metabolite was sulfonated (Table 1). This metabolite is most likely derivative of hydroxyphenylacetamide sulfate (HPAA-derivative sulfate) as the molecular ion had also a neutral loss of 90.03 Da, tentatively a loss of C3H6O3, which would match well with the proposed structural configuration [41]. HPAA is a conversion product of metabolites belonging to the benzoxazinoid class that has recently been described as a phytochemical group found in rye and wheat brans [42]. The highest concentrations of benzoxazinoids are found in germ of the grain, while the aleurone and seed coat layers are the second most abundant [43]. The gastrointestinal metabolism of benzoxazinoids is not yet described, but it has been reported that phenylacetamides, which can be derived from the benzoxazinoids are accumulating in urine [40] and plasma (Hanhineva et al., unpublished) in humans after rye rich diet. In addition to the HPAA-derivative sulfate, a further degraded form, namely 2-aminophenol sulfate (cluster 2) was also found to be excreted in urine only after addition of aleurone into the diet, and has likewise been linked with whole grain consumption as urinary biomarker [15].

The cluster 3 included also a metabolite feature with *m/z* of 232.9762 that was only detected in the urine of aleurone-receiving groups (Fragmentation pattern in Figure 7A). The compound was identified as 3,5-DHBA-sulfate based on comparison with unconjugated standard compound and on the fragmentation pattern published earlier [17]. 3,5-DHBA is derived from the phenolic phytochemical group of alkylresorcinols, a known biomarker of whole grain wheat and rye intake [44,45]. Alkylresorcinols have been shown to be mainly located in other parts of the grain than in aleurone [46]. The native aleurone used in our work has the purity degree of 85%, and it contained therefore also a small amount of other parts of the grain, which can be the source for the alkylresorcinols (1.74 mg/g, data not shown), and which may explain the urinary excretion of this biomarker.

### Relation to the findings in mice bodyweights and glucose metabolism

The physiological responses of the different aleurone preparations (A1 to A4 and commercial FA) are described in the first part of this study (Rosa N, Pekkinen J, Zavala K, Fouret G, Ayhan Kormaz A, Feillet-Coudray C, Atalay M, Hanhineva K, Mykkänen H, Poutanen K, Micard V: Enzymatic modification of wheat aleurone reduces body weight and metabolic risk factors of obesity in mice fed a high-fat diet. Submitted). The mice on a diet containing enzymatically modified aleurone preparation (A4 diet) reduced body weight (-33%), adiposity (-16%), and fasting plasma levels of leptin (-44%) (p = 0.0185, *t*-test) and insulin (-30%) (p = 0.0732, *t*-test) when compared to HF control group (Rosa N, Pekkinen J, Zavala K, Fouret G, Ayhan Kormaz A, Feillet-Coudray C, Atalay M, Hanhineva K, Mykkänen H, Poutanen K, Micard V: Enzymatic modification of wheat aleurone reduces body weight and metabolic risk factors of obesity in mice fed a high-fat diet. Submitted). Mice fed the diet supplemented with free FA had also significantly reduced fasting insulin levels (-35%) (p = 0.037, *t*-test), but no difference in the bodyweight or in the amount of adipose tissue as compared to the mice fed HF diet. In contrast, the diet with ultra-fine ground aleurone fraction (A2) resulted in increased weight gain (+9%), adiposity (+9%), and fasting plasma insulin levels (+22%) when compared to HF control group (Rosa N, Pekkinen J, Zavala K, Fouret G, Ayhan Kormaz A, Feillet-Coudray C, Atalay M, Hanhineva K, Mykkänen H, Poutanen K, Micard V: Enzymatic modification of wheat aleurone reduces body weight and metabolic risk factors of obesity in mice fed a high-fat diet. Submitted). It is interesting to note that the physiological responses considered beneficial were most pronounced in mice receiving the A4 diet, which contained mainly all of the FA in its free/soluble form.

However, according to the results from the present study the improvements in the health status of the mice fed with A4 diet cannot be explained by the content of FA and its major metabolites solely, as the beneficial effects in the FA group were limited to improved glucose metabolism, and not to other measured biomarkers including fasting leptin levels. The A4 diet contained numerous other aleurone-bound phytochemicals that were potentially released in the processing and were increased in urine of mice on the A4 diet. However, majority of these metabolites were unidentified, but those could play a role in the physiological response observed in the A4 group mice. The A4 diet presented also a high amount of soluble AX oligomers, due to the xylanase treatment, which could further induce prebiotic effects as these AX oligomers are known to be selectively fermented by the colonic microflora [47-50], and therefore the observed effects could be also mediated via modulation of microbiota composition.

## Conclusion

It was shown for the first time that changes in the structure of the wheat aleurone matrix, without altering the energy or macronutrient content, can affect the metabolic fate of various phenolic compounds and other phytochemicals. We demonstrated differential urinary metabolite profiles in a diet-induced obese mice fed chemically similar but structurally different aleurone preparations. The increase in free FA in the aleurone matrix resulting from the enzymatic processing caused increased excretion of main FA metabolites including sulfonated FA and feruloylglycine. However, when FA was bound to the insoluble arabinoxylan fibre matrix as in the case of native aleurone and cryo-ground aleurone layers, mostly microbially fermented FA metabolites were found in urine. Furthermore, the inclusion of aleurone to the diet in any form resulted in specific fingerprint on the metabolite profile including benzoxazinoid derivatives and alkylresorcinol metabolite 3,5-DHBA. Beneficial health effects, namely reduced body weight, adiposity, fasting leptin levels, and improved glucose metabolism, were observed predominantly in the mice fed the enzymatically processed aleurone, but not the pure FA group, suggesting that combined action of readily bioavailable FA and the other aleurone released metabolites are behind the bioactivities leading to these beneficial effects. Overall, the results indicate that partial depolymerization of the cell wall matrix of the wheat aleurone might be favorable for their metabolism. Therefore, in further studies structural features of grain ingredients should be considered in addition to their chemical composition, as they might explain some of the variation in human studies with this type of ingredients.

## Abbreviations

AX: Arabinoxylan; DIO: Diet-induced obesity; DF: Dietary fibre; FA: Ferulic acid; Xyl + FAE: Xylanase + Feruloylesterase; DHPPA: Dihydroxyphenylpropionic acid; HPPA: Hydroxyphenylpropionic acid; DHBA: Hihydroxybenzoic acid; DOPAC: Dihydroxyphenylacetic acid; HPAA: Hydroxyphenyl acetamide; UPLC-QTOF-MS: Ultra-performance liquid-chromatography-quadrupole time-of-flight-mass spectrometry; m/z: Mass to charge ratio; amu: Atomic mass unit.

## Competing interest

The authors declare that they have no competing interest.

## Authors’ contributions

JP, NNR, HM, KP, VM, and KH designed the research. NNR and JP conducted the animal experiment. JP was responsible for the non-targeted metabolomics analyses and for the drafting of the manuscript. JP, OS, and KH took part in the identification of the metabolites. PK was responsible for the UPLC-QTOF-MS methodology and optimizing the auto MS/MS methods. JP, NNR, HM, KP, VM, and KH contributed to the interpretation of the data. All authors read and approved the final manuscript.

## Supplementary Material

Additional file 1: Figure S1 Shows the MS/MS spectra for tentatively identified vanillic acid sulfate m/z 246.9918 with retention time 0.99 min. The identification was based on comparing the MS/MS spectra with the MS/MS spectra of standard compounds vanillic acid and DOPAC (both unconjugated), and to the suggested fragments for DOPAC sulfate and vanillic. **Figure S2.** The fragmentation pattern of tentatively identified sinapic acid sulfate with m/z 305.0386. The identification is based on matching molecular formula and the MS/MS fragmentation pattern acid found in the existing literature. Click here for file
