# Supplementary material for: Disintegration of wheat aleurone structure has an impact on the bioavailability of phenolic compounds and other phytochemicals as evidenced by altered urinary metabolite profile of diet-induced obese mice
Source: Nutr Metab (Lond). 2014 Jan 2;11:1. doi: 10.1186/1743-7075-11-1 (PMC3891979; doi:10.1186/1743-7075-11-1)
Supplement: Additional file 1: Figure S1 — Shows the MS/MS spectra for tentatively identified vanillic acid sulfate m/z 246.9918 with retention time 0.99 min. The identification was based on comparing the MS/MS spectra with the MS/MS spectra of standard compounds vanillic acid and DOPAC (both unconjugated), and to the suggested fragments for DOPAC sulfate and vanillic. Figure S2. The fragmentation pattern of tentatively identified sinapic acid sulfate with m/z 305.0386. The identification is based on matching molecular formula and the MS/MS fragmentation pattern acid found in the existing literature. [file 1743-7075-11-1-S1.pdf]

Nutrition&Metabolism

Electronic Supplementary Material

Disintegration of wheat aleurone structure has an impact on the bioavailability of phenolic compounds and other phytochemicals as evidenced by altered urinary metabolite profile of diet-induced obese mice

Jenna Pekkinen, Natalia Rosa, Otto Savolainen, Pekka Keski-Rahkonen, Hannu Mykkänen, Kaisa Poutanen, Valérie Micard, Kati Hanhineva

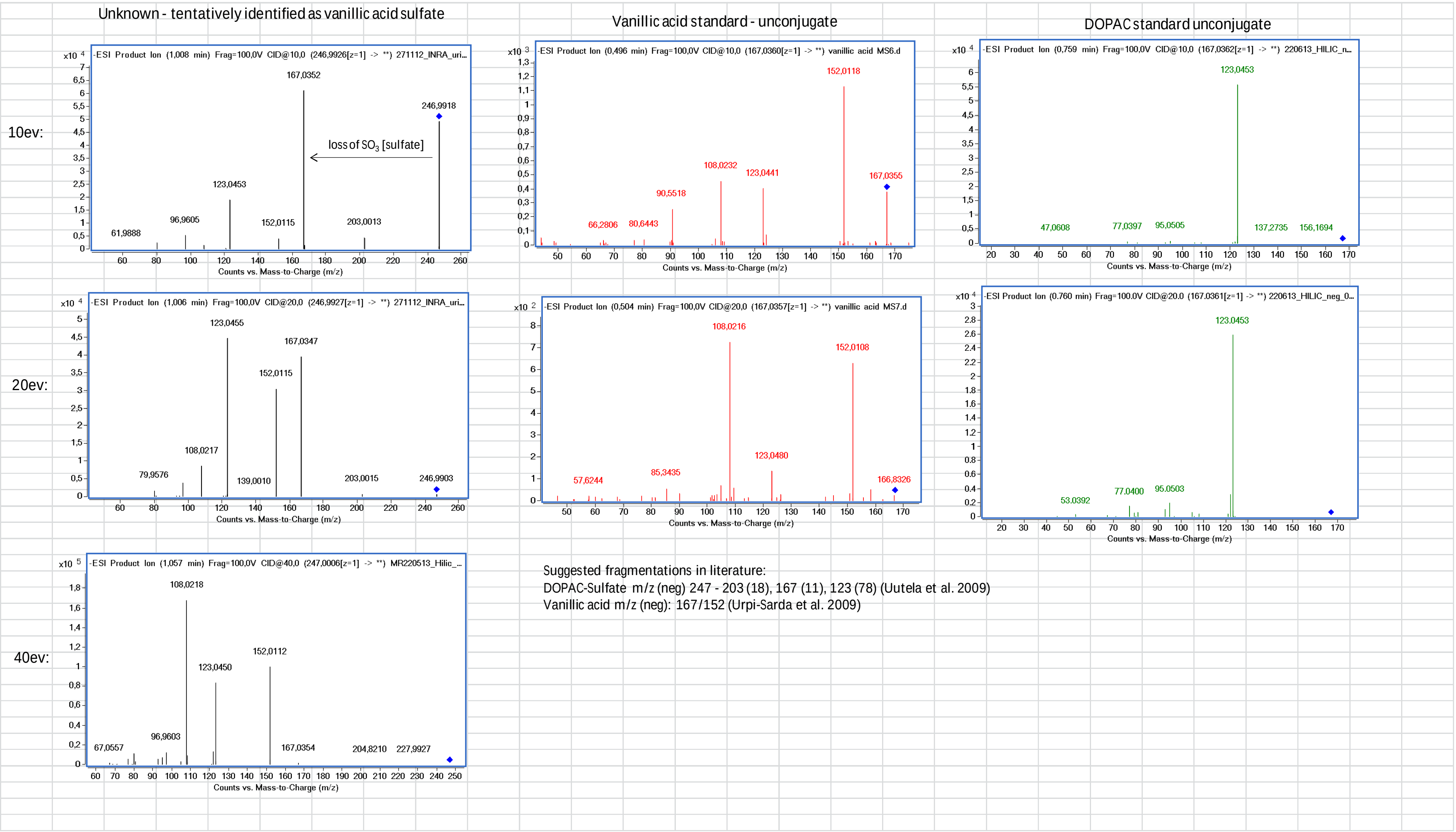

Figure S1. Shows the MS/MS spectra for tentatively identified vanillic acid sulfate m/z 246.9918 with retention time 0.99 min. The identification was based on comparing the MS/MS spectra with the MS/MS spectra of standard compounds vanillic acid and DOPAC (both unconjugated), and to the suggested fragments for DOPAC sulfate and vanillic acid found in the existing literature.

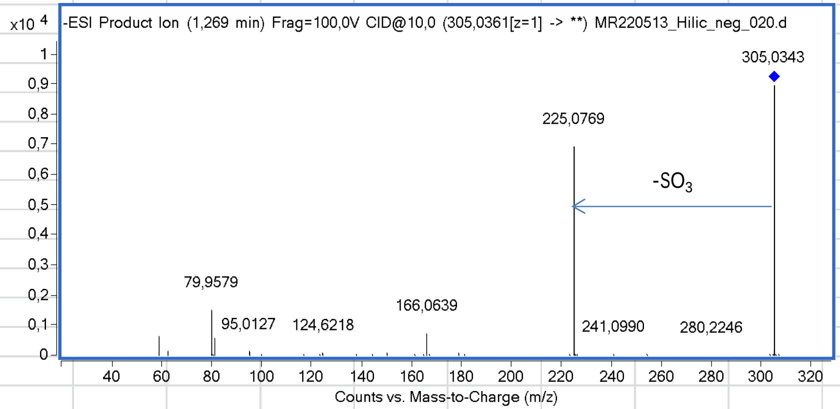

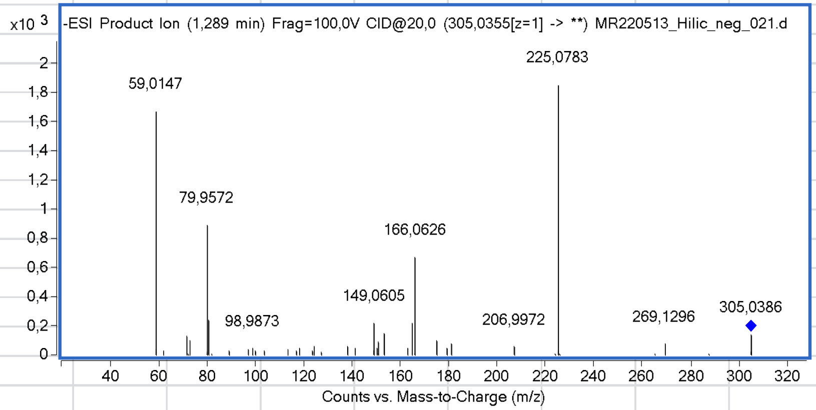

1) Molecular formula: C11H14O8S, mfg-score 98.36, diff 0.63ppm)
2) the MS/MS fragmentation created fragments 225.0783 [M-SO_3-H]$^-$, 206.9972 [M-SO_3-OH-H]$^-$, and 166.0639 [2H$^+$ more than one of the suggested fragments of sinapic acid (MID 45738)]

The metabolite is tentatively dihydrosinapic acid sulfate.

Figure S2. The fragmentation pattern of tentatively identified sinapic acid sulfate with m/z 305.0386. The identification is based on matching molecular formula and the MS/MS fragmentation pattern.
